# Children Eating

**Published:** 1877-05

**Authors:** 


### CHILDREN EATING.

When a child is observed to have little or no appetite for breakfast, sickness of some kind is impending. If in addition to this indifference for food in the morning, there is a uniform desire for a hearty supper at the close of the day, a dyspepsia for life will be founded, which will embitter many an otherwise happy hour; or some other form of chronic disease will result, which medical skill for many years, will often fail to eradicate.

This want of appetite in the morning, and this over appetite late in the day, is the creator of disease in multitudes of grown persons who have reached maturity in good health, but whose change of position, of business, or of associations, has gradually led to the perversion of nature's laws.

Young children naturally, in common with the animal creation, are greedy for breakfast, after the long abstinence of twelve hours, this is the natural arrangement, and it is wise.

Those who do not work hard, and who will have supper, "Tea," should, especially where there are children, have nothing more inviting than cold bread and butter; "Relishes," such as cake or chipped beef and the like, however "simple" they may be, are only stimulants to eating, when nature does not require any thing, and we goad her at our peril.

